# Performance of Local Light Microscopy and the ParaScreen Pan/Pf Rapid Diagnostic Test to Detect Malaria in Health Centers in Northwest Ethiopia

**DOI:** 10.1371/journal.pone.0033014

**Published:** 2012-04-20

**Authors:** Tekola Endeshaw, Patricia M. Graves, Berhan Ayele, Aryc W. Mosher, Teshome Gebre, Firew Ayalew, Asrat Genet, Alemayehu Mesfin, Estifanos Biru Shargie, Zerihun Tadesse, Tesfaye Teferi, Berhanu Melak, Frank O. Richards, Paul M. Emerson

**Affiliations:** 1 The Carter Center, Addis Ababa, Ethiopia; 2 The Carter Center, Atlanta, Georgia, United States of America; 3 Amhara Regional Health Bureau, Bahir Dar, Ethiopia; 4 Strategic Information Team, The Global Fund to Fight AIDS, Tuberculosis and Malaria, Geneva, Vernier, Switzerland; Instituto de Higiene e Medicina Tropical, Portugal

## Abstract

**Background:**

Diagnostic tests are recommended for suspected malaria cases before treatment, but comparative performance of microscopy and rapid diagnostic tests (RDTs) at rural health centers has rarely been studied compared to independent expert microscopy.

**Methods:**

Participants (N = 1997) with presumptive malaria were recruited from ten health centers with a range of transmission intensities in Amhara Regional State, Northwest Ethiopia during October to December 2007. Microscopy and ParaScreen Pan/Pf® RDT were done immediately by health center technicians. Blood slides were re-examined later at a central laboratory by independent expert microscopists.

**Results:**

Of 1,997 febrile patients, 475 (23.8%) were positive by expert microscopists, with 57.7% *P.falciparum*, 24.6% *P.vivax* and 17.7% mixed infections. Sensitivity of health center microscopists for any malaria species was >90% in five health centers (four of which had the highest prevalence), >70% in nine centers and 44% in one site with lowest prevalence. Specificity for health center microscopy was very good (>95%) in all centers. For ParaScreen RDT, sensitivity was ≥90% in three centers, ≥70% in six and <60% in four centers. Specificity was ≥90% in all centers except one where it was 85%.

**Conclusions:**

Health center microscopists performed well in nine of the ten health centers; while for ParaScreen RDT they performed well in only six centers. Overall the accuracy of local microscopy exceeded that of RDT for all outcomes. This study supports the introduction of RDTs only if accompanied by appropriate training, frequent supervision and quality control at all levels. Deficiencies in RDT use at some health centers must be rectified before universal replacement of good routine microscopy with RDTs. Maintenance and strengthening of good quality microscopy remains a priority at health center level.

## Introduction

Accurate early case detection and prompt treatment with appropriate antimalarial drugs is the major strategy for effective case management in malaria patients [1]. Correct diagnosis is also vital for the malaria prevalence and incidence indicators used to evaluate the impact of malaria control interventions [2]. A parasite based diagnostic test (microscopy or rapid diagnostic test [RDT]) is now recommended, if available, instead of presumptive treatment for all persons with suspected malaria [3]. While this recommendation has been adopted in the latest version of the Ethiopia treatment guidelines [4], diagnostic test facilities are not always available and their quality has not been comprehensively assessed or compared under routine conditions.

We previously reported two studies on ParaScreen Pf/PAN RDT in Ethiopia, one from a large household survey in mainly asymptomatic persons [5] and one from ten health centers in Amhara region [6]. ParaScreen can distinguish between a *P.falicparum* (or mixed) infection, and a non *P.falciparum* infection. The sensitivity of ParaScreen compared to expert microscopy was relatively low in the household survey [5], but it performed better for persons with suspected malaria in the health facilities in Amhara region [6]. The health facility study directly compared two RDTs, ParaScreen and ParaCheck (detects *P.falciparum* only), done by the health center technicians with the results on the same individuals by expert microscopy. The ratio of *P.falciparum* to *P.vivax* was 64% to 46%. The findings indicated that overall, ParaScreen had adequate performance of 80% sensitivity for *P.falciparum* and 74% for *P.vivax*, with 97% and 99% specificity respectively. ParaCheck also performed well for *P.falciparum* but it is not designed to detect *P.vivax,* and has been replaced with multi-species RDTs supplied to all health posts (which do not have microscopy) in Ethiopia. The higher level Health Centers and Hospitals retain the use of microscopy for malaria diagnosis.

**Figure 1:**
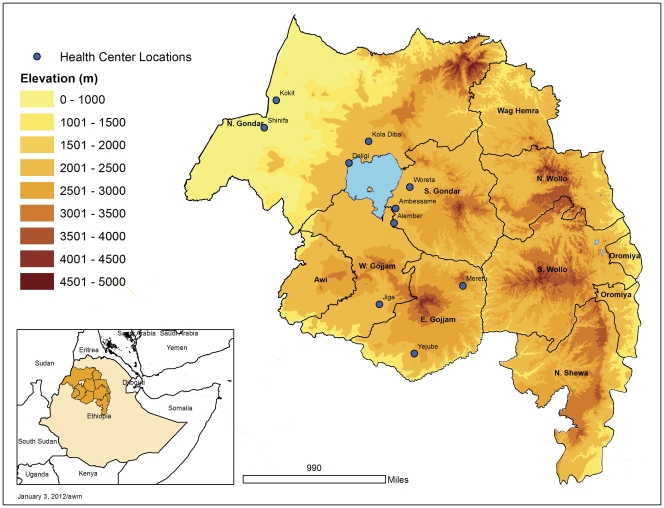
Location of health centers included in the study in Amhara National Regional State, Northwest Ethiopia.

A recent study at three health centers in Oromia region observed slightly higher sensitivity but lower specificity for *P.falciparum* by ParaScreen (85.6% and 92.4% respectively) compared to expert microscopy than we previously observed in Amhara [7]. For *P.vivax* they observed 82.5% sensitivity and 96.2% specificity with ParaScreen. Overall regardless of other parameters used for comparing the performance of three RDTs, ParaScreen performed similarly to two other tests (CareStart and ICT Combo) for *P.falciparum* but CareStart had better specificity for *P.vivax.* The slide positivity rates among patients with suspected malaria by expert microscopy were very similar in the two studies (23.8% in Amhara [6] and 23.2% in Oromia regions [7).

**Figure 2:**
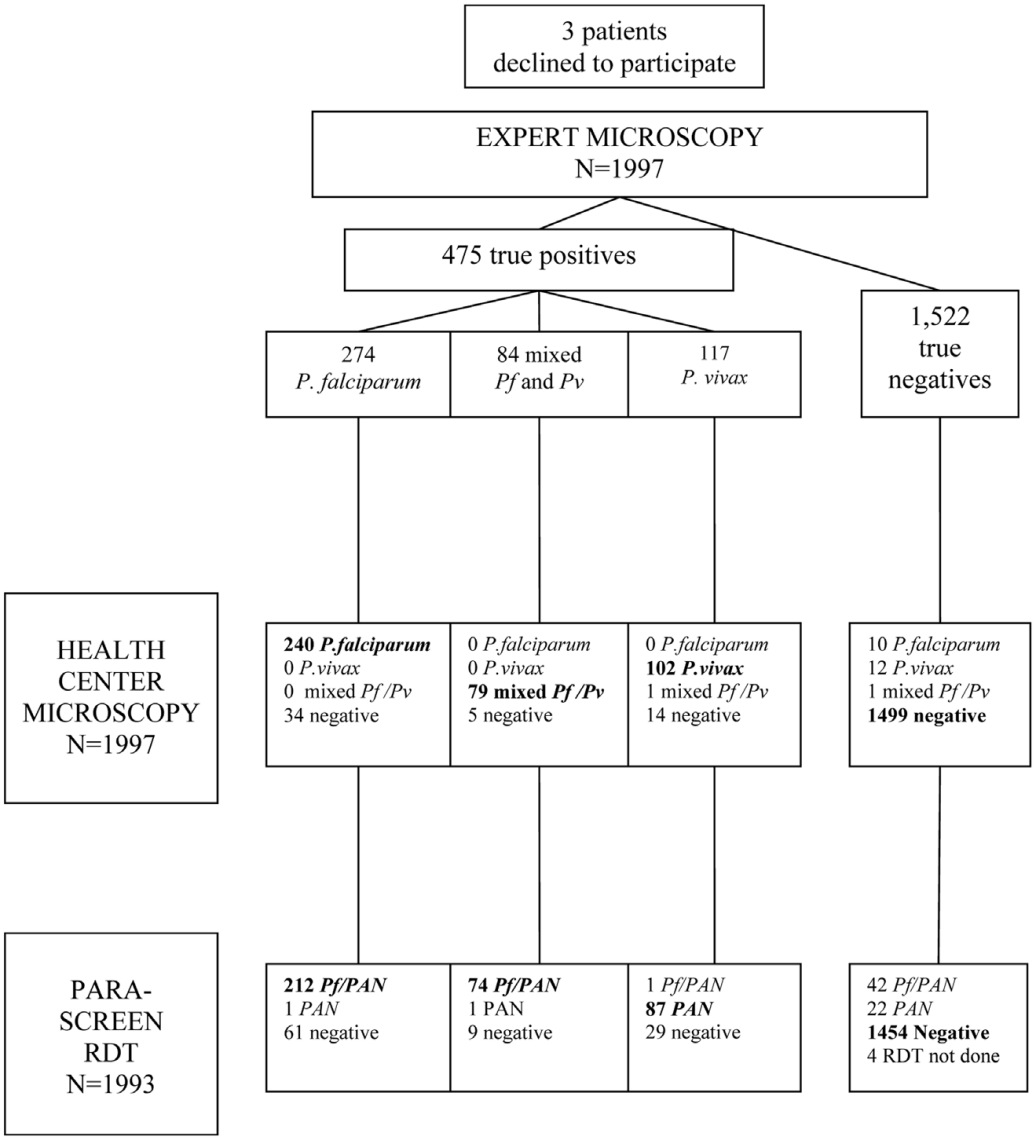
Flowchart of health center technician microscopy and ParaScreen RDT results compared to expert microscopy.

**Figure 3:**
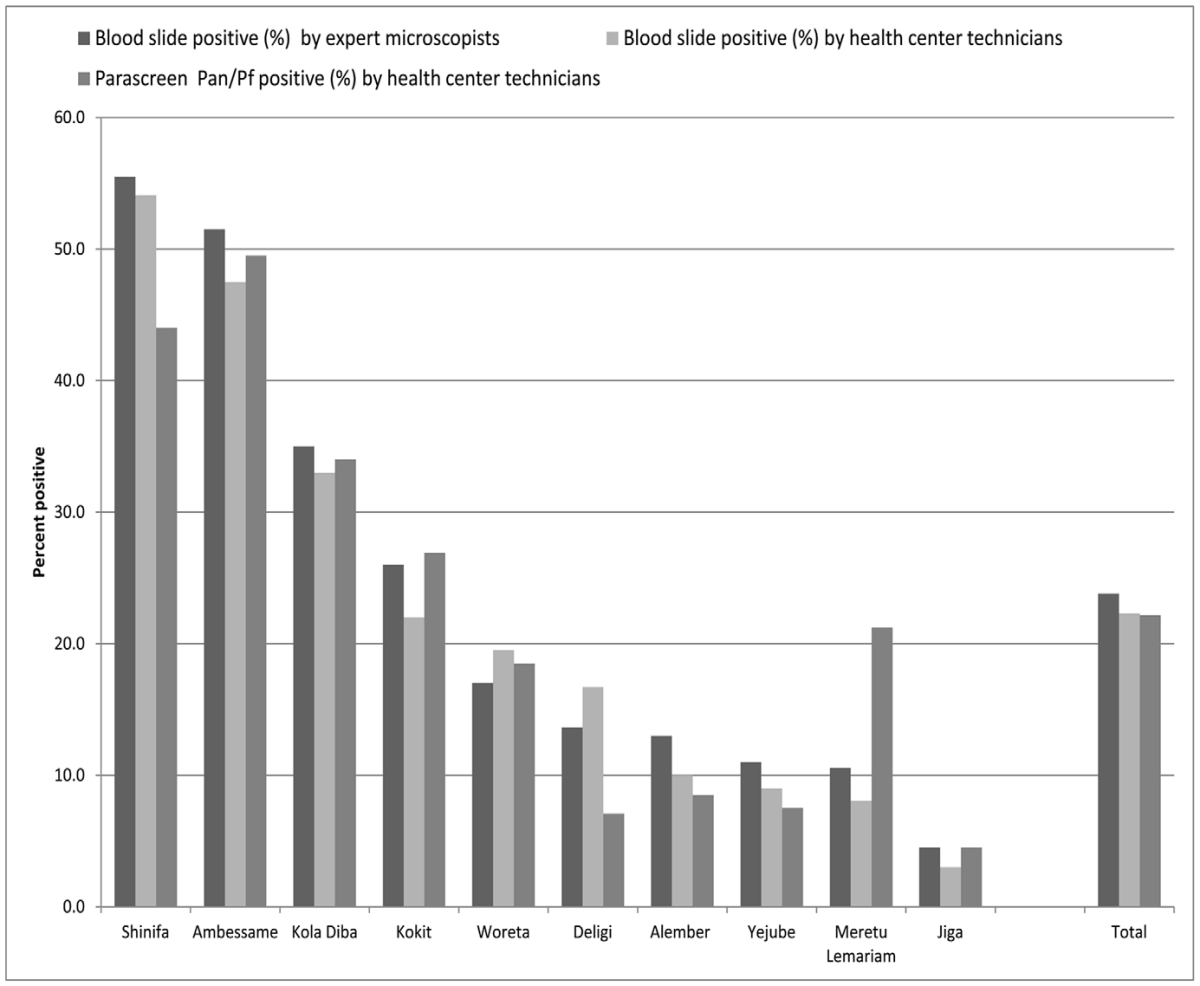
Test positive rate by health center.

**Table 1:** Prevalence of malaria by expert microscopists, by health center and species.

Name of health center	Total No. examined	No. pos Pf or mixed	No. pos Pv	No. pos any species	% positive (any species)
Shinifa	200	95	16	111	55.5
Ambessame	200	67	36	103	51.5
Kola Diba	200	62	8	70	35.0
Kokit	200	47	5	52	26.0
Woreta	200	24	10	34	17.0
Deligi	198	16	11	27	13.6
Alember	200	20	6	26	13.0
Yejube	200	6	16	22	11.0
Meretu Lemariam	199	16	5	21	10.6
Jiga	200	5	2	9	4.5
**TOTAL**	**1997**	**358**	**115**	**475**	**23.8**

Although in our previous study ParaScreen performance was acceptable overall in the Amhara health centers [6], variation was noted between health centers in the accuracy of both microscopy and RDT compared to the expert microscopists. This variation in performance at health center level is important because in Ethiopia, RDTs are routinely done at health posts (where microscopy is not available) by health extension workers, and immediate supportive supervision for these workers is expected to be provided by the cluster heath center staff at their respective catchment health posts. In addition during times of emergency, failure of microscopes and/or shortage of reagents, multispecies RDTs have to be used in the health centers, so detailed know-how on the performance of multispecies RDTs by the health center technicians is crucial. Therefore, we build on the previously reported results and conduct additional analysis with three aims:

1. To investigate the variation between health centers in the performance of the microscopists working in the health centers compared to expert microscopists;
2. To investigate further the variation between health centers in the accuracy of ParaScreen RDT performed on site, in comparison with results of expert microscopists.
3. To compare indirectly the performance of local microscopy and ParaScreen RDT for diagnosing malaria in NorthWest Ethiopia.

## Methods

### Ethical Considerations

The study protocol received ethical approval from the Emory University Institutional Review Board (IRB 00006389) and the Amhara Regional Health Bureau (Reference No. R3H5.05/1/2760). Verbal informed consent was sought from each individual and from parents of children aged under 18 years; assent was sought from children 6 to 18 years in accordance with the tenets of the Declaration of Helsinki. All positive cases were treated at their respective health centers according to the treatment guidelines for malaria infection in Ethiopia. Personal identifiers were removed from the data set before the analyses were undertaken.

### Study Settings and Population Selection

As previously described [6], the study was conducted in ten health centers (selected to cover a range of transmission intensities) in Northwest Ethiopia (Fig. 1) during the peak transmission period of malaria infection between 16^th^ Oct and 30 Dec 2007. The coordinates of each health center were recorded using a Garmin ETrex GPS unit.

In each health center the first 200 self-presenting patients of any age and either sex who qualified as clinically presumptive malaria (i.e. an axillary temperature greater than or equal to 37.5°C or history of fever in the previous 48 hours) were recruited to the study after excluding individuals with other known causes of non malarial febrile illnesses or serious illness. After obtaining informed consent demographic data were recorded on a structured questionnaire and a finger-prick blood sample taken for blood film preparation and ParaScreen RDT processing.

**Figure 4:**
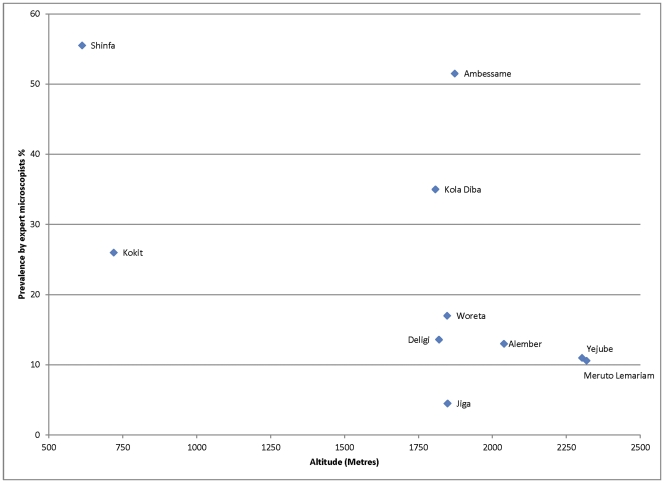
Malaria slide positivity rate in relation to altitude.

### Training

Among the ten technicians involved in this study, two held a university degree (BSc in medical laboratory technology) and the other eight held a diploma (or advanced diploma) in medical laboratory technology. Nine technicians had a minimum of five years’ experience in malarious areas and the other had two and a half years’ experience. All the technicians who participated during the training were from government health centers and had previous exposure and experience working with a monospecies malaria RDT (Paracheck Pf) that detects Pf only in their respective health centers.

**Figure 5:**
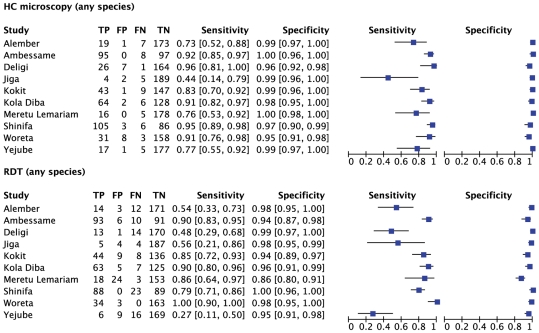
Sensitivity and specificity of local health centre microscopy and RDT compared to expert microscopy for the outcome ‘positive for any malaria species’, by health center. TP =  true positive; FP = false positive; FN = false negative; TN = true negative.

**Figure 6:**
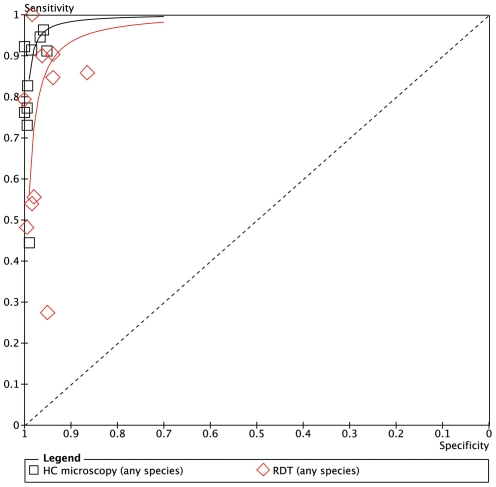
Summary receiver operator characteristic curve (SROC) for local health centre microscopy and RDT compared to expert microscopy for the outcome ‘positive for any malaria species’.

**Table 2:** Health center microscopy compared to expert microscopy: any malaria species.

Health center	Sensitivity % (95% CI)	Specificity % (95% CI)	Positive predictive value % (95% CI)	Negative predictive value % (95% CI)
Shinfa	94.6 (88.1–97.9)	96.6 (90.5–99.3)	97.2 (92.1–99.4)	93.5 (86.3–97.6)
Ambessame	92.2 (85.3–96.6)	100.0 (96.3–100)	100.0 (96.2–100)	92.4 (85.5–96.7)
Kola Diba	91.4 (82.3–96.8)	98.5 (94.6–99.8)	96.9 (89.5–99.6)	95.5 (90.5–98.3)
Kokit	82.7 (69.7–91.8)	99.3 (96.3–99.9)	97.7 (87.9–99.9)	94.2 (89.3–97.3)
Woreta	91.2 (76.3–98.1)	95.2 (90.7–97.9)	79.5 (63.5–90.7)	98.1 (94.7–99.6)
Deligi	96.3 (81.0–99.9)	95.9 (91.8–98.3)	78.8 (61.1–91.0)	99.4 (96.7–99.9)
Alember	73.1 (52.2–88.4)	99.4 (96.9–99.9)	95.0 (75.1–99.9)	96.1 (92.2–98.4)
Yejube	77.3 (54.6–92.2)	99.4 (96.9–99.9)	94.4 (72.7–99.9)	93.7 (93.7–99.1)
Meruto Lemariam	76.2 (52.8–91.8)	100.0 (97.9–100)	100.0 (79.4–100)	97.3 (93.4–99.1)
Jiga	44.4 (13.7–78.8)	98.9 (96.3–99.9)	66.7 (22.3–95.7)	97.4 (94.1–99.2)
**TOTAL**	**88.4 (85.2–91.2)**	**98.4 (97.6–98.9)**	**94.4 (91.8–96.3)**	**96.5 (95.4–97.3)**

The training, conducted for half a day at each health center, focused on technical operation of multispecies RDT (ParaScreen) based on the manufacturer’s instruction, and procedure for standard blood smear preparation. This included how to handle RDTs, how to collect blood from finger prick for both RDT and smear preparation, how to use buffer for RDT, and RDT reading and interpretation. The procedures for blood films (thin and thick) preparation, staining and species identification were briefly addressed. During training before sample collection was started, simplified and detailed standard operating procedures (SOP) on both RDT and blood slide preparation and staining were prepared and distributed to all health centers that have participated in this study. Similarly, agreement was reached with registered health officers and clinical nurses about the selection criteria of febrile patients with suspected malaria that fulfill the requirement of the study. It was also agreed with the health officers and nurses that all patients involved in the study would be treated according to the malaria treatment algorithm and national guideline of the country.

The centers were visited four times during sample collection and processing, and there was frequent telephone communication whenever there was a need to clarify study related issues or during shortage of materials to be replaced.

**Figure 7:**
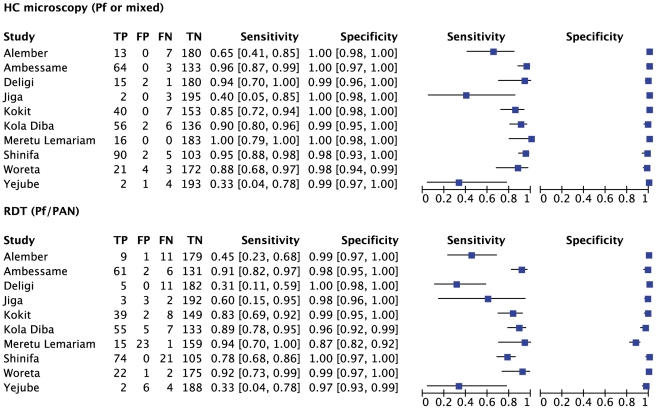
Sensitivity and specificity of local health centre microscopy and RDT compared to expert microscopy for the outcome ‘positive for *P.falciparum* or mixed infection’, by health center. TP = true positive; FP = false positive; FN = false negative; TN = true negative.

**Figure 8:**
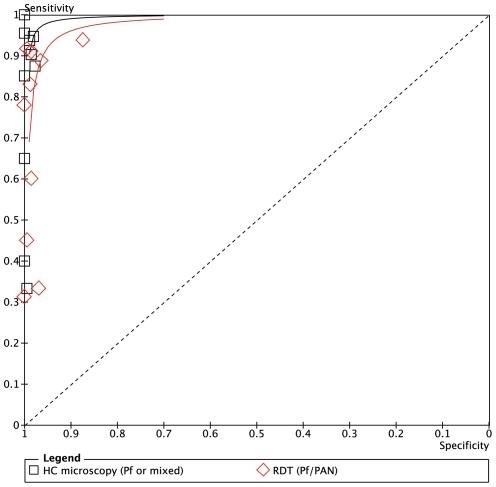
Summary receiver operator characteristic curve (SROC) for local health centre microscopy and RDT compared to expert microscopy for the outcome ‘positive for *P.falciparum* or mixed infection’.

**Table 3:** Health center microscopy compared to expert microscopy: *P.falciparum* infection only.

Health center	Sensitivity % (95%CI)	Specificity % (95%CI)	Positive predictive value % (95%CI)	Negative predictive value % (95%CI)
Shinfa	93.3 (85.1–97.8)	98.4 (94.3–99.8)	97.2 (90.3–99.7)	96.1 (91.1–98.7)
Ambessame	93.6 (82.5–98.7)	100.0 (97.6–100.0)	100.0 (91.9–100)	98.1 (94.5–99.6)
Kola Diba	89.5 (78.5–96.0)	98.6 (95.0–99.8)	96.2 (87.0–99.5)	95.9 (99.3–98.5)
Kokit	89.5 (75.2–97.1)	100.0 (97.6–100)	100.0 (89.7–100)	97.6 (93.9–99.3)
Woreta	85.7 (57.2–98.2)	98.4 (95.4–99.7)	80.0 (51.9–95.7)	98.9 (96.1–99.9)
Deligi	100.0 (66.4–100)	98.9 (96.2–99.8)	81.8 (48.2–99.7)	100.0 (98.5–100.0)
Alember	30.0 (6.7–65.3)	100.0 (98.1–100)	100.0 (29.2–100)	96.5 (92.8–98.6)
Yejube	20.0 (0.5–71.6)	99.5 (97.2–99.9)	50.0 (1.3–98.7)	97.9 (94.9–99.5)
Meruto Lemariam	100.0 (79.4–100)	100.0 (98.0–100)	100.0 (79.4–100)	100.0 (98.0–100)
Jiga	0.0	100.0 (98.1–100)	0.0	98.5 (95.7–99.7)
**TOTAL**	**87.6 (83.1–91.3)**	**99.4 (98.9–99.7)**	**96.0 (92.8–98.1)**	**98.1 (97.3–98.7)**

### Malaria Parasites Detection

#### Blood slide preparation

The finger-prick blood samples were collected by medical laboratory technicians and processed for thin and thick films according to standard WHO protocol [8], as previously described [6]. Slides were also sent for expert microscopy at The Carter Center in Addis Ababa where they were examined in blinded fashion.

#### Rapid Diagnostic Tests

Patients were tested with ParaScreen Pan/Pf® (Zephyr Biomedical systems, Verna, Goa, India) device according to the manufacture’s instruction. ParaScreen RDT had long expiry dates (6 months or more) and were stored according to the manufacturer’s recommendations (4–30°C). Tests with no band at the control line were considered invalid. Band formation on the Pan-line only was considered to be evidence of non-falciparum malaria (presumably *P. vivax* infection) whilst bands at both Pan and Pf were considered *P.falciparum* or mixed infections.

#### Statistical analysis

Statistical analysis was conducted using SPSS version 15.0 (IBM http://www-01.ibm.com/software/analytics/spss/) and RevMAN 5.1 (Review Manager (RevMan) [Computer program]. Version 5.1. Copenhagen: The Nordic Cochrane Centre, The Cochrane Collaboration, 2011). The performance of health center microscopy and of ParaScreen RDT was determined by calculating the sensitivity, specificity, positive predictive value and negative predictive value against reference laboratory microscopy as the gold standard. Sensitivity was calculated as the proportion of positive test results against true positives; specificity was calculated as a proportion of negative test results against true negatives. The positive predictive value was calculated as a proportion of true positive results among all positively reacting samples and the negative predictive was calculated as the proportion of true negative results among all negatively reacting samples. Proportions were compared using the chi-squared test. Summary receiver operator characteristic curves (SROC) were prepared in RevMAN for the two comparisons (local microscopy vs expert microscopy; RDT vs expert microscopy) and presented side by side for each of three outcomes (any malaria positive, *P.falciparum* or mixed, *P.vivax* or PAN only) by health center.

**Figure 9:**
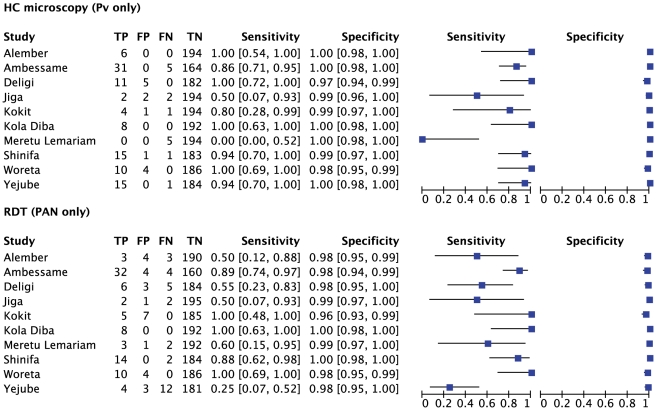
Sensitivity and specificity of local health centre microscopy and RDT compared to expert microscopy for the outcome ‘*P.vivax* or PAN only’, by health center. TP = true positive; FP = false positive; FN = false negative; TN = true negative.

**Table 4:** Health center microscopy compared to expert microscopy: *P.vivax* only.

Health center	Sensitivity % (95%CI)	Specificity % (95%CI)	Positive predictive value % (95%CI)	Negative predictive value % (95%CI)
Shinfa	93.8 (69.8–99.8)	99.5 (97.0–99.9)	93.8 (69.8–99.8)	99.5 (97.0–99.9)
Ambessame	86.1 (70.5–95.3	100.0 (97.8–100)	100.0 (88.8–100)	97.0 (93.2–99.0)
Kola Diba	100.0 (63.1–100	100.0 (98.1–100)	100.0 (63.1–100)	100.0 (98.1–100)
Kokit	80.0 (28.4–99.5)	99.5 (97.2–99.9)	80.0 (28.4–99.5)	99.5 (97.2–99.9)
Woreta	100.0 (69.2–100)	97.9 (94.7–99.4)	71.4 (41.9–91.6)	100.0 (98.0–100)
Deligi	100.0 (75.1–100)	97.3 (93.9–99.1)	68.8 (41.3–88.9)	100.0 (97.9–100)
Alember	100.0 (54.1–100.0)	100.0 (98.1–100.0)	100.0 (54.1–100.0)	100.0 (98.1–100.0)
Yejube	93.8 (69.8–99.8)	100.0 (97.0–99.9)	100.0 (78.2–100)	99.5 (97.0–99.9)
Mertu-Lemariam	0.0	100.0 (98.1–100)	0.0	97.5 (94.2–99.2)
Jiga	50.0 (6.8–93.2)	98.9 (96.4–99.9)	50.0 (6.8–93.2)	98.9 (96.4–99.9)
**TOTAL**	**82.2 (79.7–92.6)**	**99.3 (98.8–99.6)**	**88.7 (81.5–93.8)**	**99.2 (98.7–99.6)**

**Table 5:** Rapid Diagnostic Test (Pf/PAN or PAN) compared to expert microscopy: any malaria species.

Health center	Sensitivity % (95%CI)	Specificity % (95%CI)	Positive predictive value % (95%CI)	Negative predictive value % (95%CI)
Shinfa	79.3 (71.7–86.8)	100.0 (95.9–100.0)	100.0 (95.9–100.0)	79.5 (72.0–86.9)
Ambessame	90.3 (84.6–96.0)	93.8 (89.0–98.6)	93.9 (89.2–98.6)	90.1 (84.3–95.9)
Kola Diba	90.0 (83.0–97.0)	96.2 (98.2–99.5)	92.6 (86.4–98.6)	94.7 (90.9–98.5)
Kokit	84.6 (74.8–94.4)	93.9 (90.1–97.8)	83.0 (72.9–93.1)	94.6 (90.9–98.2)
Woreta	100.0 (89.7–100.0)	98.2 (94.8–99.6)	91.9 (78.1–98.3)	97.6 (93.9–99.3)
Deligi	48.1 (29.3–67.0)	99.4 (96.8–99.9)	92.9 (66.1–100.0)	92.4 (88.6–96.2)
Alember	53.8 (34.7–73.0)	98.3 (95.0–99.6)	82.4 (56.6–96.2)	93.4 (89.9–97.0)
Yejube	27.3 (8.7–45.9)	94.9 (91.7–98.2)	40.8 (15.2–64.8)	94.9 (91.7–98.2)
Meruto Lemariam	85.7 (70.7–100.7)	86.5 (81.5–91.5)	42.9 (27.9–57.8)	98.1 (95.9–100)
Jiga	55.6 (23.1–88.0)	97.9 (95.9–99.9)	55.6 (23.1–88.0)	97.9 (95.9–99.9)
**TOTAL**	**79.4 (75.5–82.9)**	**95.7 (94.6–96.7)**	**85.3 (81.6–88.5)**	**93.7 (92.4–94.9)**

**Table 6:** Rapid Diagnostic Test (Pf/PAN) compared to expert microscopy: *P.falciparum* and mixed infections.

Health center	Sensitivity % (95%CI)	Specificity % (95%CI)	Positive predictive value % (95%CI)	Negative predictive value % (95%CI)
Shinfa	77.9 (69.9–86.2)	100.0 (96.5–100)	100.0 (95.1–100)	83.3 (76.8–89.8)
Ambessame	91.0 (84.2–97.9)	98.5 (94.7–99.8)	96.8 (89.0–99.6)	95.6 (92.2–99.0)
Kola Diba	88.7 (80.8–96.6)	96.4 (93.3–99.5)	91.7 (84.7–98.7)	95.0 (91.4–98.6)
Kokit	83.0 (72.2–93.7)	98.7 (95.4–99.8)	95.1 (83.5–99.4)	95.0 (91.6–98.4)
Woreta	91.7 (73.0–98.9	99.4 (96.9–99.8)	95.7 (78.1–99.9)	98.9 (95.9–99.8)
Deligi	31.3 (8.5–54.0)	100.0 (97.9–100)	100.0 (47.8–100)	94.3 (91.0–97.6)
Alember	45.0 (23.2–66.8)	99.4 (96.9–99.9)	90.0 (55.5–99.7)	94.2 (90.9–97.5)
Yejube	33.3 (4.4–71.1)	96.9 (94.5–99.3)	25.0 (5.0–55.0)	97.9 (95.9–99.9)
Meruto Lemariam	93.8 (69.8–99.8)	87.4 (82.6–92.2)	39.5 (23.9–55.0)	99.4 (96.6–99.9)
Jiga	60.0 (14.7–94.7)	98.5 (95.6–99.7)	50.0 (10.0–90.0)	98.9 (96.3–99.9)
**TOTAL**	**79.6 (75.1–83.7)**	**97.4 (96.5–98.1)**	**86.7 (82.8–90.4)**	**95.6 (94.5–96.6)**

**Figure 10:**
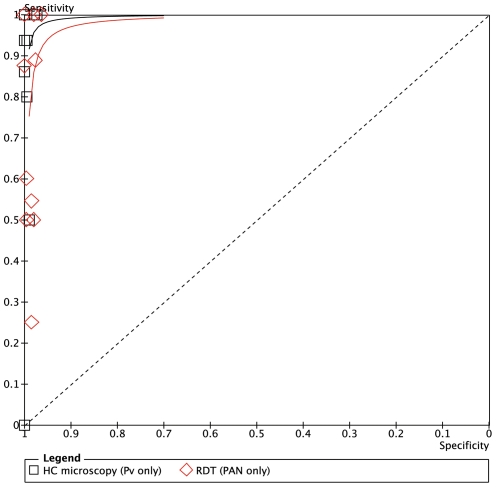
Summary receiver operator characteristic curve (SROC) for local health centre microscopy and RDT compared to expert microscopy for the outcome ‘*P.vivax* or PAN only’.

**Table 7:** Rapid Diagnostic Test (PAN only) compared to expert microscopy: *P.vivax* only.

Health center	Sensitivity % (95%CI)	Specificity % (95%CI)	Positive predictive value % (95%CI)	Negative predictive value % (95%CI)
Shinfa	87.5 (61.7–98.5)	100.0 (98.0–100.0)	100.0 (76.8–100)	98.9 (96.2–99.8)
Ambessame	88.9 (78.6–99.2)	97.6 (95.2–99.9)	88.9 (78.6–99.2)	97.6 (95.2–99.9)
Kola Diba	100.0 (63.1–100)	100.0 (98.1–100.0)	100.0 (63.1–100)	100.0 (98.1–100.0)
Kokit	100.0 (47.8–100)	96.4 (93.8–99.0)	41.7 (13.8–69.6)	100.0 (98.1–100)
Woreta	100.0 (69.2–100)	97.9 (95.9–99.9)	71.4 (47.8–95.1)	100.0 (98.0–100)
Deligi	54.5 (25.1–84.0)	98.4 (95.4–99.7)	66.7 (35.9–97.5)	97.4 (95.1–99.6)
Alember	50.0 (10.0–90.0)	97.9 (95.9–99.9	42.9 (6.2–79.5)	98.5 (95.5–99.7)
Yejube	25.0 (3.8–46.2)	98.4 (95.3–99.7)	57.1 (20.5–93.8)	93.8 (90.4–97.2)
Meruto Lemariam	60.0 (14.7–94.7)	99.5 (97.2–99.9)	75.0 (19.4–99.4)	99.0 (96.3–99.8)
Jiga	50.0 (10.0–90.0)	99.5 (97.2–99.9)	66.7 (9.4–99.2)	98.9 (96.4–99.8)
**TOTAL**	**74.4 (65.5–81.9)**	**98.6 (97.9–99.1)**	**76.3 (67.4–83.8)**	**98.4 (97.7–98.9)**

## Results

Locations of the health centers are shown in Figure 1. Out of 2000 recruited patients, 1997 febrile cases were examined for malaria parasites by blood slide microscopy (198 to 200 per health center). Out of these, 56.2% were males and the remaining were females. The age range was 8 months to 85 years with a mean of 20.7 years. Of the 1997 persons tested by slide, 1993 samples were also examined by ParaScreen RDT at the health centers. During supervisory visits to the health centers, it was observed in some health centers that the technicians were overloaded with different laboratory work due to high flow of outpatients seeking treatment and laboratory tests.

The results for all the health centers combined are shown in the flow chart in Figure 2. By expert microscopy (the gold standard), 23.8% of the 1997 patients tested were positive for malaria parasites, with a range from 4.5% to 55.5% by health center (Table 1 and Figure 3). Results for health center microscopy were overall 22.3% positive (N = 1997) with a range of 3.0 to 54.1%; and for ParaScreen RDT 22.2% positive (N = 1993) with a range of 4.5 to 49.5%. These differences between expert microscopists, health center microscopists and RDTs in overall percent positive are not statistically significant. However, they mask significant variation at the health center level.

Altitudinal variation in relation to malaria slide positivity is shown in Figure 4. In general there was a declining trend of positivity rate with altitude, with the lowest rates being observed at altitudes higher than 2000 meters above sea level, but there were two health centers between 1750 and 2000 meters above sea level with high slide positivity rates (Ambessame with 51.5% and Kola Diba with 35% slide positivity rate). The possible explanation for high malaria positive rate in these two health centers at high altitude could be that the majority of the patients were from the catchment villages of lower altitude of known malarious areas.

### Health Center Microscopy Compared to Expert Microscopy

The majority of infections (57.7%) detected by expert microscopists were *P.falciparum* only, with 24.6% *P.vivax* and 17.7% mixed infections (Table 1 and Figure 2). The overall ratio of *P.falciparum* to *P.vivax* (1.78∶1 for the experts) was comparable for the health center microscopists (1.69∶1).

By individual health center, overall percent positive was not significantly different between health center and expert microscopists in any health center. However the general concordance in slide positive rate mentioned above and shown for the total sample in Figure 3 does not represent the complete picture, since there was not complete overlap in the positives or the species identified by the two sets of microscopists (Figure 2).

Figure 5 expresses the sensitivity and specificity for the outcome of malaria positive (any species) at each health center against expert microscopy, and Figure 6 shows the results in Summary Receiver Operator characteristic (SROC) format. The positive and negative predictive values are given in Table 2.

The overall sensitivity of microscopy for any malaria species by the health center microscopists was 88.4% (95% CI 85.2–91.2) and the specificity was 98.4% (95% CI 97.6–98.9). In the six health centers with highest prevalence (Shinifa, Ambessame, Kola Diba, Woreta, Deligi and Alember), sensitivity was greater than 90% in five of them and above 80% in Kokit (Figure 5). In three of the medium transmission areas (Meruto-Lemariam, Yejube and Alember), sensitivity of 70 to 80% was observed. Notably, Figure 5 shows very poor sensitivity by the health centre microscopist of 44.4% (95% CI 13.7–78.8) at Jiga health center, which had the lowest positivity rate of all the centers. Specificity was above 95% at all the centers.

Figures 7 and 8 show the equivalent results for the comparison of health center microscopy versus expert microscopy for *P.falciparum* or mixed infections. Positive and negative predictive values are given in Table 3. Sensitivity for *P.falciparum* was above 80% in 7 of the 10 centers, and specificity was 98% or higher in all (Figure 7). Two sites (Yejube and Jiga) had relatively low sensitivity for *P.falciparum* (<60%).

For *P.vivax* results are shown in Figs. 9 and 10. Sensitivity and specificity are shown graphically in Figure 9 while positive and negative predictive values are in Table 4. For *P.vivax* (Figure 9), the majority of the sites had sensitivity above 80% and specificity was very good; only one site (Meruto Lemariam) showed very poor sensitivity for *P.*vivax.

### ParaScreen Rapid Diagnostic Test Compared to Expert Microscopy

For any malaria species (Figure 6), the overall sensitivity of RDTs was 79.4%. Only 3 of the health centers (Ambessame, Kola Diba and Woreta) had sensitivity over 90%), two (Kokit and Meruto Lemariam) were between 80 and 90%, one (Shinfa) was 79% and the other four were below 60% sensitivity. Specificity was very good overall with the exception of Meruto Lemarian with 86% specificity. The SROC curves for the outcomes of malaria positive (any species) are shown in Figure 7 while positive and negative predictive values are in Table 5.

The proportion of positives (any species) detected by RDT was significantly lower than the expert microscopists at two health centers: Shinifa (44% RDT vs 55.5% expert microscopy, Chi-sq = 5.29, p = 0.021) and Deligi (7.1% RDT vs 13.6% expert microscopy, Chi-sq = 4.60, p = 0.032), while percent positive was higher by RDT at Meruto Lemariam (21.1% RDT vs. 13.2% expert microscopy, Chi-sq = 4.49, p = 0.034). The others were not significantly different.

For *P.falciparum* or mixed infection sensitivity and specificity are shown in Figure 7, SROC in Figure 8 and PPV and NPV given in Table 6. Four of the health centers (Deligi, Alember, Yejube and Jiga) with lower prevalence (see Table 1) performed poorly with RDTs (Figure 7). Meruto Lemariam was the exception among the health centers with low prevalence in achieving very good sensitivity for *P.falciparum*, although at the expense of specificity.

For *P.vivax* (PAN only by RDT), Figures 9 and 10 and Table 7 present the results. The same four low prevalence centers mentioned above (Deligi, Alember, Yejube and Jiga) performed very poorly (<60%) on RDT sensitivity, and Meruto Lemarian had only 60% sensitivity. The two centers with highest overall prevalence (Shinfa and Ambessame) had 88–89% sensitivity while the other 3 were over 90%.

### ParaScreen Rapid Diagnostic Test Compared to Health Center Microscopy

Only indirect comparison is possible because the same technicians conducted both tests in each health center, which compromised the blinding. The relative accuracies of health centre microcopy and RDT for each of three outcomes (any species, Pf or mixed, Pv) are shown in the Summary ROC curves in Figures 6, 8 and 10 respectively. In each case the RDT predicted curve lies to the right and below (less accurate) that for HC microscopy.

The five sites with highest prevalence were relatively consistent in giving good or very good performance for both microscopy and RDT compared to expert microscopy. However overall, the performance of RDT was not as good as health center microscopy, and it was particularly poor in the five sites with lower prevalence. As expected, the RDTS performed in general less well for *P.vivax* than *P.falciparum.*

## Discussion

Rapid diagnostic tests are being strongly promoted for wider use to ensure that all suspected malaria cases receive a diagnosis before treatment. Most RDT studies have tested whether RDTs are as accurate as expert microscopy, and these previous studies were mostly designed to assess the performance of the tests *per se*, rather than their accuracy in routine use. There have been few evaluations of the accuracy of RDTs compared to the *status quo* of routine health center microscopy, or of variation in performance of both routine microscopy and RDT between sites. The results of such studies point to differences in strict application of knowhow gained during training and previous work experience in malarious areas (for both methods) as well as storage or other possible factors that affect the correct use of RDTs. Even if RDTS are not as good as expert microscopy, in some cases they may be better than routine microscopy. In this study we address this issue indirectly by examining the performance of both routine microscopy and RDTs as performed in ten rural health centers, compared to the gold standard of expert microscopy.

Overall, microscopists in ten rural health centers in Amhara region, Northwest Ethiopia showed fair to very good performance compared to expert microscopy, with the exception of the health center with the lowest prevalence of 4.5% among suspected malaria cases. One other health center did badly with *P.vivax* slides only. Microscopists in health centers in these study sites of Northwest Ethiopia are performing to a standard higher than has been observed in some other malaria endemic areas [9]. However there are still some gaps and inconsistencies in microscope capacity, and lack of a standardized quality control system for diagnostics, as has been observed by others [10].

For RDTs, there was large variation between sites in the performance, with generally lower performance than for local microscopy, when each is compared to expert microscopy. Four of the ten sites (of the five with less malaria) performed very poorly on RDT sensitivity in general, and the other was very poor for *P.vivax*. Even one of the sites with high prevalence demonstrated only a fair level of sensitivity with RDTs. Sensitivity of the test (unlike positive predictive value) should not be affected by prevalence. A decrease in positive predictive value for RDT in one site with lower prevalence was also observed in Uganda [11].

During supervisory visits it was noted that although the technicians were observed to be proficient in performing the tests according to standard operating procedures, they were overloaded with the many other lab tests they are expected to perform in addition to malaria diagnosis. Under real world conditions, when pressed with large numbers of patients, they may use rapid staining methods and skimp on slide examination time or number of fields to be examined (especially if densities are low) just to satisfy the clients. Low prevalence in an area with few requests for malaria diagnosis gives the technician limited ability to maintain his or her skills in parasite identification by microscopy, or to practice reading and interpreting faint positive RDT tests. More quality control checks and frequent refresher trainings are needed in low incidence areas, or as malaria incidence declines due to extensive control efforts.

Overall our results demonstrate slightly lower sensitivity with RDTs than has been observed in Ethiopia and elsewhere [7], [10]. The low sensitivity with ParaScreen in some sites means that cases are being missed while high false positive rates means that persons without malaria (and possibly with other infections) are getting treated for malaria in some sites. These findings suggest that there are deficiencies in strict application of training materials, lack of previous skill in performing multispecies RDTs, and/or possible problems in RDT handling conditions in some sites, in addition to large demands on technicians’ time for other lab tests. Where no adequate and standard malaria microscopy exists (for example in health posts staffed by Health Extension Workers in moderate to high malarious areas), this study supports the introduction of multispecies RDTs for improvement of diagnosis of malaria, provided that they are accompanied by adequate training on procedure and limitations of the tests, as well as continual supervision and overall quality control mechanisms. However, microscopy in rural health centers remains the local ‘gold standard’ and should not be neglected for refresher training and supervision especially where problems are identified in particular centers as in this study.
